# Bcl-XL Inhibits Membrane Permeabilization by Competing with Bax

**DOI:** 10.1371/journal.pbio.0060147

**Published:** 2008-06-10

**Authors:** Lieven P Billen, Candis L Kokoski, Jonathan F Lovell, Brian Leber, David W Andrews

**Affiliations:** 1 Department of Biochemistry and Biomedical Sciences, McMaster University, Hamilton, Ontario, Canada; 2 Department of Medicine, McMaster University, Hamilton, Ontario, Canada; University of California San Francisco, United States of America

## Abstract

Although Bcl-XL and Bax are structurally similar, activated Bax forms large oligomers that permeabilize the outer mitochondrial membrane, thereby committing cells to apoptosis, whereas Bcl-XL inhibits this process. Two different models of Bcl-XL function have been proposed. In one, Bcl-XL binds to an activator, thereby preventing Bax activation. In the other, Bcl-XL binds directly to activated Bax. It has been difficult to sort out which interaction is important in cells, as all three proteins are present simultaneously. We examined the mechanism of Bax activation by tBid and its inhibition by Bcl-XL using full-length recombinant proteins and measuring permeabilization of liposomes and mitochondria in vitro. Our results demonstrate that Bcl-XL and Bax are functionally similar. Neither protein bound to membranes alone. However, the addition of tBid recruited molar excesses of either protein to membranes, indicating that tBid activates both pro- and antiapoptotic members of the Bcl-2 family. Bcl-XL competes with Bax for the activation of soluble, monomeric Bax through interaction with membranes, tBid, or t-Bid-activated Bax, thereby inhibiting Bax binding to membranes, oligomerization, and membrane permeabilization. Experiments in which individual interactions were abolished by mutagenesis indicate that both Bcl-XL–tBid and Bcl-XL–Bax binding contribute to the antiapoptotic function of Bcl-XL. By out-competing Bax for the interactions leading to membrane permeabilization, Bcl-XL ties up both tBid and Bax in nonproductive interactions and inhibits Bax binding to membranes. We propose that because Bcl-XL does not oligomerize it functions like a dominant-negative Bax in the membrane permeabilization process.

## Author Summary

During development and under stress, cells can become committed to die via programmed cell death (apoptosis). In most cases, the permeabilization of the outer mitochondrial membrane is a key component of this commitment. The membrane permeablization step is both positively and negatively regulated by members of the Bcl-2 family of proteins. One member of this protein family with only a BH3 region, such as tBid, activates another family member, Bax, causing it to form large complexes that generate membrane-spanning pores, hence making the membrane permeable. Antiapoptotic members of the Bcl-2 family, such as Bcl-XL, are structurally similar to Bax but inhibit the membrane permeabilization process by an unknown mechanism. Two mutually exclusive models have been proposed to explain how the Bcl-2 family is operating: one states that Bcl-XL binds to tBid, thereby preventing Bax activation, while the second suggests that Bcl-XL binds directly to activated Bax. It has been difficult to sort out which interaction is important in cells, where multiple members of all three protein families are present simultaneously. Here, we describe an in vitro system containing the three recombinant proteins and the use of mutagenesis to selectively remove individual interactions. We show that Bcl-XL inhibits Bax by competing with it for binding to membranes, tBid, and activated Bax. Because Bcl-XL does not form pores, it inhibits apoptosis by acting as if it is a dominant-negative version of Bax.

## Abstract 3

Bcl-XL and Bax are structurally similar members of the Bcl-2 family of cell-death-related proteins, and they compete for binding to membranes, as well as to Bcl-2 family member tBid and activated Bax. Unlike Bax, Bcl-XL is unable to oligomerize and form pores in membranes, so it inhibits membrane permeabilization--a key step during commitment to apoptosis--by functioning like a dominant-negative Bax.

## Introduction

Apoptosis is a form of programmed cell death important for development and tissue homeostasis, and its deregulation has been implicated as the cause of many disease processes. Apoptosis may be initiated by a developmental program and by many diverse forms of cell stress. A critical feature of metazoan apoptosis is the permeabilization of intracellular organellar membranes that leads to the egress of intraorganellar components that activate the proteases responsible for cell death. Bcl-2 is the founding member of a family that includes members that either prevent (e.g., Bcl-2 or Bcl-XL) or promote (e.g., Bax or Bak) the membrane permeabilization that leads to apoptosis. Another large subgroup of the Bcl-2 family (BH3-only proteins; e.g., tBid) initiates apoptosis through binding to Bax and/or Bcl-XL. Even though Bcl-XL and Bax are structurally similar, experiments with protein fragments and peptides or full-length protein in the absence of membranes has led to the elaboration of models in which the functional relevance of binding partners for Bcl-XL differ. The direct activation [1,2] or hierarchical model [3] states that Bcl-XL and other antiapoptotic Bcl-2 family proteins inhibit apoptosis primarily through interactions with a subclass of BH3-only proteins termed “activators”, preventing them from activating Bax and Bak. The indirect activation model [4,5] proposes that the function of all BH3-only proteins is to displace the inhibitory Bcl-XL from inherently active Bax or Bak. Thus, these two models differ primarily in whether Bax/Bak is constituively active or requires activation and which interactions are crucial to the antiapoptotic function of Bcl-XL. However, there is also considerable overlap between these two competing models: both models recognize that Bcl-XL directly binds to and inhibits a proapoptotic Bcl-2 family protein that is directly involved in membrane permeabilization, while other proapoptotic Bcl-2 family proteins indirectly induce apoptosis by binding to Bcl-XL and preventing this function. Moreover, some authors postulate that both models may be relevant in different cell types or in the same cell type under different circumstances (e.g., normal versus cancerous) [2,6]. Our previous results have demonstrated the importance of dynamic interaction of Bcl-2 family members with membranes [7–9]. On the basis of these data and evidence from the literature, we have proposed a model termed “embedded together”, in which interaction of these proteins with each other changes after binding to the membrane as this causes conformational changes that alter and/or expose new binding surfaces [10,11]. Accordingly, a key feature for resolving differences in the components and mechanisms of physiologically relevant interactions between Bcl-2 family members is to examine these interactions in membranes.

To examine the molecular mechanism of membrane permeabilization for full-length Bcl-XL and Bax, we used recombinant proteins and measured permeabilization of both liposomes and mitochondrial outer membranes in vitro. Recombinant tBid was used as an activator protein. Our results demonstrate that Bcl-XL and Bax, despite having opposite effects on apoptosis, share many functionally similar features: in the absence of an activator, neither protein bound tightly to membranes, whereas the addition of membrane-bound tBid to Bax or Bcl-XL recruited a similar molar excess of the soluble protein to membranes. However, after binding to membranes, only Bax formed large oligomers and permeabilized the membrane. Bcl-XL competes with Bax for binding to both tBid and membrane-bound Bax as well as for binding to membranes. In all cases, interaction of Bax with Bcl-XL is nonproductive for membrane permeabilization, presumably because Bcl-XL does not oligomerize. Thus, we propose that Bcl-XL functions similarly to a dominant-negative Bax in tBid-initiated membrane permeabilization.

## Results

To inhibit membrane permeabilization, Bcl-XL might inhibit any or all of the interactions of Bax and its activator with each other or with membranes. There are many potential activators of Bax, including but not limited to the BH3-only proteins Bid, Bim, and Puma as well as other non-Bcl-2 family proteins such as p53 [12,13] and Bif-1 [14,15]. While studies using cells or animals in which specific components of the apoptotic pathway have been eliminated have contributed substantially to our understanding, it would be technically difficult if not impossible to create mouse stains with all of the relevant genes knocked out. Hence, precisely which step(s) in the process are inhibited by Bcl-XL as well as the molecular mechanism of inhibition can be best determined by using a cell-free system in which each step can be examined individually. To eliminate complications from all other known and unknown proteins, metabolites, and post-translational modifications that may contribute additional levels of regulation, we used a cell-free system composed of highly purified full-length recombinant proteins, without N- or C-terminal tags, and as a source of membranes either liposomes with mitochondria-like composition [16] or subcellular fractions containing mitochondria. As a representative activator protein, we used the caspase-8 cleavage product of Bid (tBid) to activate Bax. This protein drives Bax/Bak-dependent permeabilization of mitochondria [17,18] and also binds directly to antiapoptotic proteins such as Bcl-XL and Bcl-2 [19].

**Figure 1:**
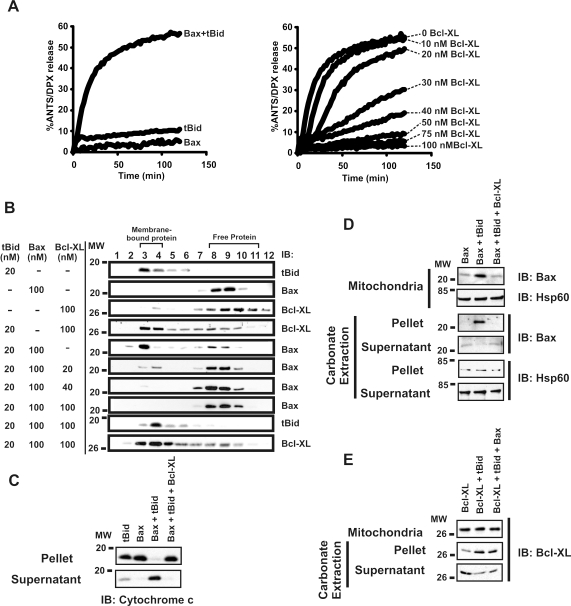
Bax and tBid Cooperate To Induce Liposome Permeabilization, Which Is Inhibited by Bcl-XL (A) Liposomes encapsulated with ANTS and DPX were incubated with 100 nM Bax, 20 nM tBid, or both (left panel) or with 100 nM Bax, 20 nM tBid, and the indicated concentrations of Bcl-XL (right panel). Membrane permeabilization was assayed by an increase of ANTS fluorescence. (B) Liposome binding of tBid, Bax, and Bcl-XL. The proteins, at the indicated concentrations, were incubated with liposomes. Membrane-bound proteins were separated from soluble proteins by Sepharose CL-2B gel filtration chromatography. Individual fractions were analyzed by immunoblotting (IB) using Bid, Bax, or Bcl-XL antibodies, as indicated. (C) Mitochondria from *bak* knockout mice were incubated with tBid (20 nM), Bax (100 nM), and Bcl-XL (100 nM), as indicated. Permeabilization was assayed by pelleting the mitochondria and analyzing both the pellet (P) and the supernatant (S) fractions by immunoblotting using an α-cytochrome c antibody. (D and E) Mitochondria were incubated with the indicated proteins (concentrations as in (C)) and the levels of (D) Bax or (E) Bcl-XL assayed in the mitochondrial pellet. Integration of Bax or Bcl-XL into mitochondrial membranes was assayed by carbonate extraction, using Hsp60 (a soluble matrix protein) as a control.

The large increase in fluorescence that accompanies the release of the fluorophore/quencher pair 8-aminonaphthalene 1,3,6-trisulfonic acid (ANTS)/*p*-xylene-bis-pyridinium (DPX) from liposomes was used to measure Bax-dependent membrane permeabilization and its inhibition by Bcl-XL [7]. As seen previously, single addition of recombinant Bax (100 nM) or tBid (20 nM) to liposomes had little effect, but in combination the two proteins caused an increase in fluorescence due to membrane permeabilization (Figure 1A). This result is consistent with previous observations that tBid-activated Bax caused permeabilization of mitochondria in cells [19], isolated mitochondria [8,9], and liposomes [16].

### Bcl-XL Prevents Membrane Binding by Bax

The earliest step in the process leading to membrane permeabilization that could be inhibited by Bcl-XL is binding of tBid or Bax to the membrane. In incubations of 20 nM tBid and liposomes, tBid bound effectively to liposomes as assayed by gel filtration chromatography (Figure 1B). Without tBid, Bax (100 nM) did not bind to liposomes, as expected from results seen in vitro and in cells [20]. However, when the two proteins were added together, 20 nM tBid caused most of the Bax to bind liposomes. The stoichiometry of this interaction confirms that one tBid molecule can directly or indirectly recruit multiple Bax molecules to membranes [21]. The resulting membrane permeabilization released ANTS/DPX and larger encapsulated fluorophores (Figure S1A) as well as fluorescent proteins of similar mass to the proteins released from the intermembrane space of mitochondria during apoptosis (unpublished data).

**Figure 3:**
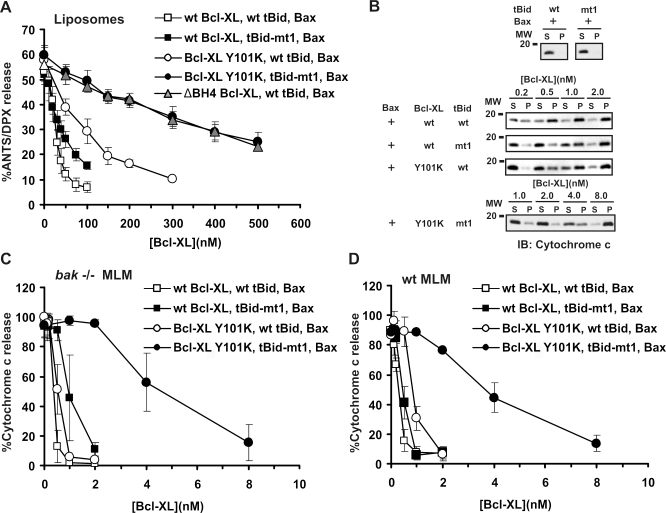
Bcl-XL Binds Both tBid and Bax To Prevent Membrane Permeabilization (A) Liposomes encapsulated with ANTS and DPX were incubated with 100 nM Bax, 20 nM tBid or tBid-mt1, and increasing concentrations of Bcl-XL, Bcl-XL Y101K, or Bcl-XL ΔBH4. Membrane permeabilization was assayed as in Figure 1A and is presented as percentage of ANTS/DPX release mean ± standard deviation for at least three independent experiments. (B) Mitochondria isolated from *bak* knockout mouse livers (*bak* –/– MLM) were incubated with 200 nM Bax, 250 pM tBid or tBid-mt1, and increasing concentrations of Bcl-XL or Bcl-XL Y101K, as indicated. Permeabilization was assayed by pelleting the mitochondria and analyzing both the pellet (P) and the supernatant (S) fractions by immunoblotting using an α-cytochrome c antibody. (C) Results from experiments as in (B) quantified as percentage of cytochrome c release mean ± standard deviation for at least three independent experiments. (D) Mitochondria isolated from wild-type mouse livers (wt MLM) were assayed as in (B) except that the concentration of tBid was reduced 10-fold to 25 pM. Permeabilization was quantified as in (C).

Addition of recombinant Bcl-XL inhibited tBid/Bax-mediated liposome permeabilization in a concentration-dependent manner (Figure 1A) with an IC_50_ of ∼25 nM at the 2 h end point. Furthermore, the shape of the inhibition curve (Figure S1B) suggests that Bcl-XL inhibits ANTS/DPX release competitively. At all effective concentrations of Bcl-XL, membrane binding by Bax was efficiently inhibited (Figures 1B and 3), as has been observed previously in cells [22] and for mitochondria [18]. While Bax membrane binding was inhibited, Bcl-XL had no effect on tBid binding to membranes (Figure 1B). These results argue that Bcl-XL inhibits membrane permeabilization by preventing the binding of Bax, but not tBid, to membranes.

To confirm that Bcl-XL inhibited tBid-induced binding of Bax to biological membranes, mitochondria purified from mouse liver (MLM) of *bak* –/– mice were assayed. MLM from *bak* –/– mice also lack Bax as it is a cytoplasmic protein in liver. Like liposomes, these mitochondria were resistant to tBid, as outer mitochondrial membrane (OMM) permeabilization, measured by the release of cytochrome c, required the addition of both tBid and Bax (Figure 1C). As seen with liposomes, Bcl-XL inhibited tBid/Bax-mediated cytochrome c release. To assess the binding of recombinant Bax to mitochondria, the organelles were incubated with or without tBid or tBid and Bcl-XL, pelleted by centrifugation, and analyzed by immunoblotting (Figure 1D). The addition of tBid increased the level of membrane-bound Bax and caused the integration of Bax into the OMM, as assessed by resistance to carbonate extraction, while the addition of Bcl-XL inhibited these effects, confirming our results obtained with liposomes. As expected, tBid was sufficient to induce permeabilization of OMM isolated from wild-type mice (as these mitochondria contained Bak), and Bcl-XL inhibited this permeabilization (unpublished data).

The correlation between tBid concentration (20 nM) and the IC_50_ of ∼25 nM Bcl-XL for inhibition of liposome permeabilization is consistent with other published models suggesting Bcl-XL sequestration of Bax/Bak activators (in this case tBid) as one mechanism by which Bcl-XL could inhibit membrane binding by Bax.

### Membrane-Bound tBid Recruits Excess Bcl-XL to Membranes, Where It Binds tBid and Bax

**Figure 4:**
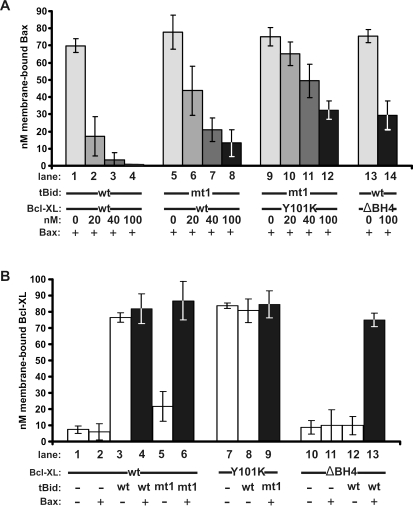
Bcl-XL Binding to Membranes and Bcl-XL-Mediated Inhibition of Bax Membrane Binding Are Mediated by Interactions with tBid and Bax (A) Quantification of Bax binding to membranes. Bax (100 nM) and tBid (20 nM) or tBid-mt1 were incubated with liposomes in the presence of increasing concentrations of Bcl-XL, Bcl-XL Y101K, or Bcl-XL ΔBH4. Membrane-bound protein was separated from soluble protein by Sepharose CL-2B gel filtration chromatography and quantified by immunoblotting. Results are presented as mean ± standard deviation for at least three independent experiments. (B) Quantification of Bcl-XL and Bcl-XL mutant protein binding to membranes. Bcl-XL, Bcl-XL Y101K, or Bcl-XL ΔBH4 (100 nM) was incubated with the indicated proteins and analyzed as above. Results are presented as mean ± standard deviation for at least three independent experiments. Black bars indicate experiments directly comparable between (A) and (B).

Alone, Bcl-XL showed minimal liposome binding, consistent with cytoplasmic or loosely membrane-bound localizations reported for Bcl-XL in live cells [23,24] (Figure 1B). However, 20 nM tBid caused migration of ∼80 nM Bcl-XL to liposomes both when the two proteins were added together (Figure 1B, quantified in Figure 4B) and when tBid was bound to liposomes before Bcl-XL was added (unpublished data). The stoichiometry of this interaction indicates that, similar to the effect on Bax, one tBid molecule recruits multiple Bcl-XL molecules. When MLM from *bak* –/– mice were used as the membrane source, the addition of tBid markedly increased the levels of membrane-integrated Bcl-XL. As a result, the Bcl-XL, which was almost entirely peripherally attached in the absence of tBid, integrated in the OMM in the presence of tBid (Figure 1E).

**Figure 2:**
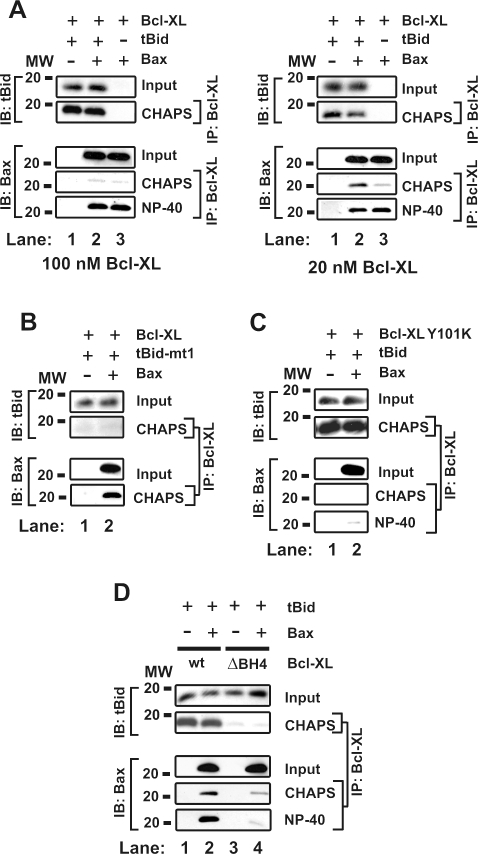
Bcl-XL Binds to Membrane-Bound tBid and Bax (A) Bax (100 nM) and/or tBid (20 nm) were incubated with 100 nM Bcl-XL (left panel) or 20 nM Bcl-XL (right panels) and liposomes. Samples were immunoprecipitated (IP) in either 2% CHAPS or 0.2% NP-40, as indicated, using an antibody with the indicated specificity and immunoblotted (IB) for the indicated protein. (B–D) Mutations prevent the binding of Bcl-XL to tBid, Bax, or both. (B and D) Bcl-XL (20 nM), or the indicated Bcl-XL mutants, (C) Bcl-XL Y101K or (D) Bcl-XL ΔBH4, were incubated with (C and D) 20 nM tBid or (B) tBid-mt1 with or without Bax (100 nM) and with liposomes. Immunoprecipitations and immunoblotting were performed as in (A).

**Figure 6:**
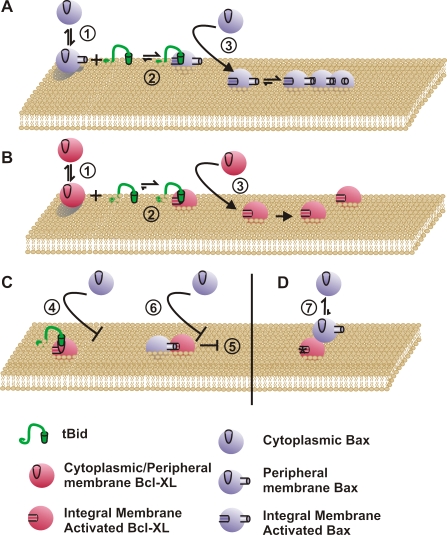
Bcl-XL Functions Like a Dominant-Negative Bax (A) Cytoplasmic Bax undergoes a conformational change after interacting with membranes (step 1). Interaction of this peripheral membrane (indicated by the shadow) Bax with membrane-bound tBid causes a further conformational change such that Bax integrates in the membrane in an oligomerization competent form (step 2). Conversely, cytoplasmic Bax may interact with other activator proteins to integrate into membranes, or spontaneously active Bax molecules may integrate into the membrane without binding an activator protein. A single tBid molecule activates multiple peripheral membrane Bax molecules, and/or the activated integral membrane Bax recruits more cytoplasmic Bax to the membrane (autoactivation, step 3). Bax oligomerizes. (B) Bcl-XL exists in a cytoplasmic and/or a peripheral membrane-bound form (step 1). Membrane-bound tBid triggers membrane binding and activation of Bcl-XL (step 2). One tBid molecule can mediate the membrane binding and activation of multiple Bcl-XL molecules (step 3). Bcl-XL does not oligomerize. (C) Membrane-bound Bcl-XL sequesters tBid and thereby prevents the activation of Bax (step 4). Bcl-XL binds to membrane-bound Bax, preventing Bax oligomerization (step 5) and the recruitment of further peripheral Bax by autoactivation (step 6). In steps 4–6, Bcl-XL functions as a dominant-negative Bax. (D) Bcl-XL inhibits the conformational change of Bax (step 7, indicated as a change in equilibrium) that is elicited by peripheral membrane binding (step 1 in (A)).

When both proteins were in the membranes, Bcl-XL bound to tBid, as assessed by co-immunoprecipitation. This interaction was not dependent on the detergent used to solubilize the liposomes (unpublished data), and at concentrations of 100 nM Bcl-XL and 20 nM tBid the interaction was not affected by the addition of Bax (Figure 2A, left panel, lanes 1 and 2). In the absence of membranes, interaction between Bcl-XL and tBid could not be detected by co-immunoprecipitation (unpublished data). Therefore, sequestering membrane-bound tBid represents one mechanism whereby Bcl-XL could inhibit apoptosis (Figure 6C, step 4). The recruitment of multiple Bcl-XL molecules by a single molecule of membrane-bound tBid increases the likelihood that tBid will be sequestered by Bcl-XL, thereby increasing how effectively Bcl-XL competes with Bax for tBid binding. Furthermore, when Bcl-XL prevented tBid/Bax-mediated membrane permeabilization (Figure 1A), Bcl-XL was overwhelmingly membrane-bound (Figure 1B). Thus, contrary to models that propose that membrane integration would inactivate Bcl-XL [25], our results suggest that tBid is *required* to cause a conformational change in Bcl-XL that allows it to insert into membranes where it may inhibit tBid (Figure 6B).

Although sequestration of membrane-bound tBid by Bcl-XL appears to account for its antiapoptotic function in both liposomes and MLM, we sought to determine whether Bcl-XL also could interact stably with Bax. When tested in the absence of tBid, a stable interaction between Bax and Bcl-XL was not detected (Figure 2A, left panel, lane 3), suggesting that Bcl-XL does not sequester Bax in solution. However as expected, co-immunoprecipitation of Bcl-XL and Bax was observed in control experiments where membranes were solubilized with the nonionic detergent NP-40, known to induce a conformational change in Bax required for heterodimerization with Bcl-XL that is also seen in cells when apoptosis is induced [20,26].

When tested in the presence of tBid (20 nM) and membranes, we did not detect an interaction between Bcl-XL (100 nM) and Bax (100 nM) (Figure 2A, left panel, lane 2). Rather, Bcl-XL out-competed Bax for binding to both tBid and membranes. In these incubations, Bcl-XL may not bind Bax because they are not in the same compartment; Bcl-XL is almost completely membrane-bound while Bax is soluble (Figure 1B). Therefore, the same experiment was performed using 20 nM Bcl-XL, a concentration at which Bcl-XL competes less efficiently with Bax for binding to tBid, allowing some Bax to become membrane-bound. Under these conditions, Bcl-XL marginally inhibited membrane permeabilization (Figure 1A) and coprecipitated with Bax in CHAPS buffer (Figure 2A, right panel, lane 2). The addition of Bax also reduced the amount of tBid bound to Bcl-XL (compare lanes 1 and 2), suggesting that under conditions where Bcl-XL cannot fully out-compete Bax for access to tBid the presence of membrane-bound Bax provides a second target for binding by Bcl-XL (Figure 6C, step 5).

Taken together, our results indicate that Bcl-XL and Bax compete for binding to tBid and that Bcl-XL prevents membrane permeabilization by forming stable heterodimers with the membrane-bound forms of both tBid and Bax. However, because both interactions are present when Bcl-XL is active, it is possible that one particular interaction contributes more to the antiapoptotic function of Bcl-XL than the other, as has been proposed alternately by two competing models of apoptosis [3,4].

### Binding of Membrane-Bound Bcl-XL to tBid and Activated Bax Each Contribute Significantly to Inhibition of Membrane Permeabilization

To assess the relative contributions of Bcl-XL binding to tBid and Bax to antiapoptotic function, these interactions were selectively removed through mutagenesis. A tBid mutant with substitution of two residues within the BH3 domain (M97A/D98A of murine Bid, denoted tBid-mt1) does not bind stably to Bcl-XL but binds to Bax [27]. Consistent with this previous report, in our assay system tBid-mt1efficiently recruited Bax to membranes where Bax was activated (unpublished data), but tBid-mt1 did not coprecipitate with Bcl-XL (Figure 2B). Thus, tBid-mt1 allows the selective removal of the Bcl-XL–tBid interaction. Conversely, a mutation within the BH3 binding pocket of Bcl-XL (Y101K) prevents stable binding to Bax [28]. As expected, Bcl-XL Y101K bound tBid but did not bind to activated Bax (Figure 2C), even in the presence of NP-40 detergent. Therefore, using Bcl-XL Y101K allows the selective removal of Bcl-XL–Bax binding. Combining these two mutants creates a situation where Bcl-XL does not bind stably to either tBid or Bax (unpublished data). As a negative control, we used Bcl-XL with a deletion of the BH4 domain (amino acids 4–24) that has been reported to remove binding to both Bax and the BH3-only protein Bad [29]. In our assay, ΔBH4 Bcl-XL did not coprecipitate tBid and bound very inefficiently to activated Bax (Figure 2D). Addition of the Y101K point mutation to ΔBH4 Bcl-XL to further remove Bax binding did not reduce this residual interaction (unpublished data), indicating that it is nonspecific. Therefore, these mutants behave similarly in live cells and in our cell-free system.

By assay of ANTS/DPX release from liposomes for different combinations of these proteins, the residual function of Bcl-XL can be measured in the absence of stable interaction with tBid, Bax, or both. The relative effects of each mutant were tested by examining the concentration-dependent inhibition of tBid/Bax-mediated liposome permeabilization by Bcl-XL (Figure 3A). When tBid was replaced by tBid-mt1, Bcl-XL continued to inhibit membrane permeabilization and bound Bax activated by tBid-mt1 (Figure 2B). This indicates that a significant amount of Bcl-XL activity does not require Bcl-XL–tBid binding, as has been suggested previously by some [30] but not other models [3,31,32].

Removal of the Bcl-XL–Bax interaction by using Bcl-XL Y101K decreased the activity of Bcl-XL somewhat (Figure 3A), consistent with previously published results [33]. Nevertheless, the protein offered significant protection from tBid/Bax-induced dye release from liposomes. Thus, the loss of tBid or Bax binding to Bcl-XL can be compensated by the other binding interaction. The combination of tBid-mt1 and Bcl-XL Y101K or ΔBH4 Bcl-XL (situations in which Bcl-XL binds stably to neither tBid nor Bax) greatly reduced the activity of Bcl-XL but did not eliminate it completely. To address the possibility that this remaining activity was the result of residual Bcl-XL–tBid or Bcl-XL–Bax interactions at elevated concentrations of Bcl-XL, we examined the effects of the tBid-mt1 and Bcl-XL Y101K mutations on Bcl-XL–tBid and Bcl-XL–Bax interactions by co-immonoprecipitation at Bcl-XL concentrations higher than those shown in Figure 2 (Figure S2). To detect the interactions of Bcl-XL with tBid-mt1 as well as Bcl-XL Y101K with Bax required elevated concentrations of Bcl-XL and/or prolonged immunoblot exposures compared to the wild-type proteins, suggesting that these residual interactions either are nonspecific or contribute very little to the remaining antiapoptotic activity seen with the tBid-mt1/Bcl-XL Y101K combination. To test this possibility further, we combined the tBid-mt1/Bcl-XL Y101K and ΔBH4 Bcl-XL mutations. This combination of mutations (tBid-mt1 with ΔBH4 Bcl-XL Y101K) did not further diminish Bcl-XL function (unpublished data), suggesting that this remaining activity does not involve stable protein–protein interactions. This residual function of Bcl-XL is addressed below.

The effects of these proteins also were assessed for the regulation of cytochrome c release from isolated mitochondria (Figure 3B–D). By the use of *bak* –/– MLM (Figure 3B, quantified in Figure 3C), Bcl-XL inhibited tBid/Bax-induced membrane permeabilization in a dose-dependent manner, as was seen in liposomes. Substitution of tBid-mt1 or Bcl-XL Y101K for the respective wild-type proteins only slightly reduced the prevention of cytochrome c release by Bcl-XL. Using these two mutants together markedly reduced but did not eliminate the inhibition of cytochrome c release by Bcl-XL, similar to the residual activity noted with liposome permeabilization. When wild-type MLM that contained BAK were used, it was necessary to reduce the concentration of tBid 10-fold to 25 pM to prevent it from activating sufficient Bak to cause OMM permeabilization. At this concentration of tBid, cytochrome c release required the addition of Bax (unpublished data), and the activity of Bcl-XL was similar to that seen with *bak* –/– MLM (Figure 3D). In these incubations, substitution of the wild-type proteins with tBid-mt1, Bcl-XL Y101K, or both showed similar effects to those seen in *bak* –/– MLM and liposomes (Figure 3C). Thus, in contrast to widely promulgated models for the antiapoptotic mechanism of Bcl-XL, our results with purified proteins in the presence of relevant membrane targets indicate that Bcl-XL binding to Bax and tBid are both functionally relevant, but neither is paramount.

### Membrane-Bound Bcl-XL Inhibits Bax Binding to Membranes Via Multiple Mechanisms

Although our results indicate that Bcl-XL inhibits tBid-mediated activation of Bax by sequestering tBid in a stable complex, it is unclear how Bcl-XL inhibits Bax binding to membranes. For example, Bcl-XL might inhibit Bax binding to membranes by preventing it from interacting with tBid. Alternatively or in addition, Bcl-XL might directly inhibit Bax binding to membranes. To determine the mechanism(s) involved, we measured Bax liposome binding by gel filtration chromatography for reactions containing the different mutant proteins (Figure 4A).

The absence of a stable interaction between tBid-mt1 and Bcl-XL significantly decreased Bcl-XL-mediated inhibition of Bax binding to membranes at all tBid concentrations assayed. Consequently, significantly more Bax bound to membranes in reactions containing tBid-mt1 compared to otherwise identical reactions containing tBid (*p* < 0.005 for 100 nM Bcl-XL, *p* < 0.05 for 40 nM Bcl-XL, *p* < 0.1 for 20 nM Bcl-XL). However, contrary to the prediction of some models [34], Bcl-XL sequestering of tBid is only a contributing factor, as in the absence of tBid binding Bcl-XL still dramatically reduced Bax binding to membranes (Figure 4A, compare lanes 5–8).

It has been suggested that the multi-BH-region proapoptotic proteins Bax and Bak autoactivate after tBid (or another BH3-only protein) initiates the process and that autoactivation is inhibited by Bcl-2 [21,35]. In our system, one consequence of Bax autoactivation would be recruitment of soluble Bax to membranes. Therefore, in the absence of Bcl-XL/tBid heterodimerization, Bcl-XL may inhibit recruitment of Bax by binding to membrane-bound Bax and inhibiting Bax autoactivation. To address this possibility, we substituted Bcl-XL with the Bax-binding-deficient Bcl-XL Y101K and assayed Bax binding to liposomes in the presence of tBid-mt1 (Figure 4A, lanes 9–12). In reactions containing Bcl-XL Y101K, significantly more Bax bound membranes (*p* < 0.005 for 100 nM Bcl-XL, *p* < 0.05 for 40 nM Bcl-XL, *p* < 0.1 for 20 nM Bcl-XL), indicating that the interaction of Bcl-XL and Bax inhibits further Bax binding to membranes. To determine whether the Bcl-XL/Bax heterodimer also prevented the subsequent oligomerization of Bax, we examined oligomerization by cross-linking. In these experiments, the cross-linker was added to reactions containing an equal amount of membrane-bound Bax in the absence or presence of Bcl-XL (Figure S3). In these reactions, membrane-bound Bcl-XL inhibited Bax oligomerization, as detected by cross-linking concomitant with inhibition of dye release from liposomes. Taken together, these results suggest that, when bound to Bcl-XL, Bax function is neutralized, both in recruitment of other Bax molecules through autoactivation and in oligomerization to permeabilize membranes (Figure 6C, step 6).

Similar to results obtained by examining liposome permeabilization, there is a residual activity of the tBid-mt1/Bcl-XL Y101K combination that prevented Bax binding to membranes even in the absence of a stable interaction of Bcl-XL Y101K with either tBid or Bax. This activity also was observed for a ΔBH4 mutant of Bcl-XL (Figure 4A, lanes 13, 14).

At the onset of apoptosis in cells, Bcl-XL binds to membranes [23]. The addition of tBid is sufficient to trigger Bcl-XL to bind to membranes in vitro (Figure 1B). Because the membrane appears to be the active locus, we used the mutant versions of Bcl-XL and tBid to examine the importance of stable binding to tBid or Bax for Bcl-XL to bind tightly to membranes (Figure 4B). In a negative control experiment without other added proteins, less than 10 nM of the added Bcl-XL (100 nM) bound to membranes (Figure 4B, lane 1). Addition of Bax to the incubation did not increase membrane binding by Bcl-XL (lane 2). As noted above (Figure 1B), a substoichiometric amount of tBid (lane 3) caused ∼80 nM Bcl-XL to bind to membranes, suggesting that each tBid molecule recruited three to four Bcl-XL molecules. Membrane binding by Bcl-XL was reduced greatly when tBid was substituted by tBid-mt1, indicating that stable Bcl-XL–tBid binding can recruit Bcl-XL (lane 5). However, the addition of Bax and tBid-mt1 (lane 6) caused the near complete recruitment of Bcl-XL (∼85 nM membrane-bound). Under these conditions, the concentration of membrane-bound, activated Bax is only ∼15 nM (Figure 4A, lane 8), yet the concentration of membrane-bound Bcl-XL increases by ∼60 nM above that seen with tBid-mt1, indicating that similar to tBid each activated Bax molecule recruits about four Bcl-XL molecules. Thus, like tBid, tBid-activated Bax recruits both soluble Bax and Bcl-XL. Surprisingly, when both soluble Bax and Bcl-XL are present, it appears that activated Bax recruits Bcl-XL more efficiently than it recruits Bax. Therefore, another way that Bcl-XL inhibits recruitment of Bax is by competing with it for activated Bax on the membrane.

The Bax-binding-deficient mutant Bcl-XL Y101K directly and efficiently binds liposomes (Figure 4B, lanes 7–9). This is presumably due to the location of this mutated residue in the BH3 binding pocket suggested to house the C-terminal membrane anchor of soluble Bcl-XL based on structural similarity with Bax [36] or to bind the C-terminal membrane anchor of an another Bcl-XL molecule as a cytoplasmic homodimer [33]. In either case, spontaneous binding of Bcl-XL Y101K to membranes may be due to displacement of the C-terminal membrane anchor as a result of the Y101K mutation.

The functional importance of membrane binding by Bcl-XL is supported further by our observations that when Bcl-XL inhibits membrane permeability in assays containing tBid and Bax almost all of the Bcl-XL is membrane-bound and is in stoichiometric excess over membrane-bound tBid and Bax (Figure 4A and 4B, black bars), even when using mutants that prevent heterodimerization with one or both of the binding partners. Consistent with a role for membrane binding, removing the C-terminal tail that mediates membrane binding impaired but did not abolish Bcl-XL function (Figure S4), similar to the results obtained in cells using the same mutant [37]. However, removal of the C-terminal tail in the context of the tBid-mt1 and Bcl-XL Y101K mutations completely abolishes the remaining activity of Bcl-XL. It is possible therefore that the antiapoptotic function of Bcl-XL that is independent of stable interactions with tBid and Bax requires that Bcl-XL is membrane-bound. In that case, the excess Bcl-XL bound to membranes in the presence of tBid or activated Bax could contribute to this function of Bcl-XL (see below).

Taken together, our results suggest that Bcl-XL prevents recruitment of Bax to membranes by multiple mechanisms (Figure 6C). These include: competition with Bax for binding to and thereby sequestering tBid, inhibition of Bax autoactivation by competing with soluble Bax for binding membrane-bound Bax, and an unidentified mechanism requiring membrane-bound Bcl-XL.

### Membrane-Bound Bcl-XL Prevents a Membrane-Induced Reversible Bax Conformational Change

**Figure 5:**
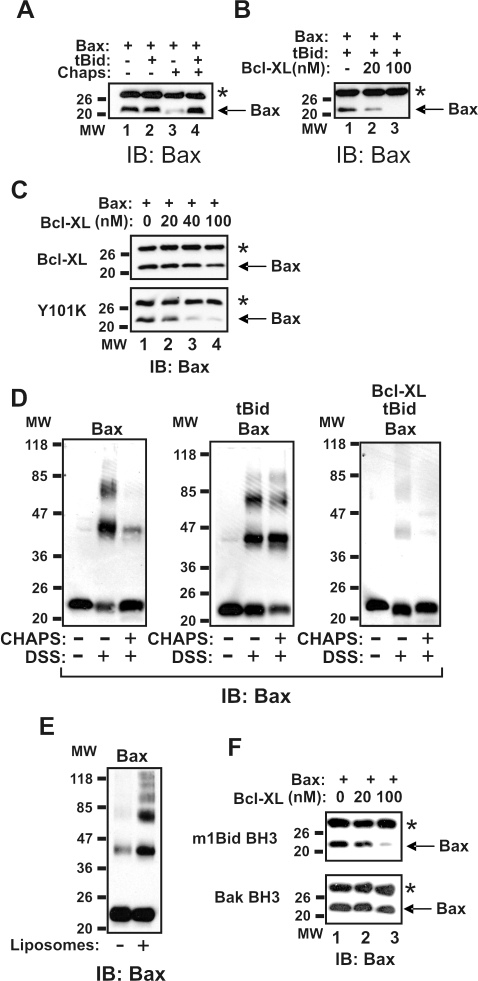
Membrane-Bound Bcl-XL Inhibits the Liposome-Induced Conformational Change in Bax (A) Bax (100 nM) was incubated in the presence of liposomes and in the absence or presence of tBid (20 nM). Conformation-altered Bax was immunoprecipitated using the 6A7 antibody with or without the addition of 2% CHAPS to solubilize the liposomes prior to immunoprecipitation and analyzed by immunoblotting using an α-Bax antibody. The asterisk denotes the light chain of the 6A7 antibody. (B) Bax was incubated in the presence of liposomes, tBid, and increasing concentrations of Bcl-XL. Immunoprecipitations and immunoblotting were performed as in (A) without the addition of 2% CHAPS. (C) Bax was incubated for 2 h with liposomes (without tBid) at increasing concentrations of Bcl-XL or Bcl-XL Y101K. Immunoprecipitations and immunoblotting were performed as in (B). (D and E) Bcl-XL inhibits liposome-induced cross-linking of Bax. (D) Bax (100 nM) was incubated with liposomes for 2 h either alone (left panel), with 20 nM tBid (middle panel), or with 20 nM tBid and 100 nM Bcl-XL (right panel). Cross-linking with DSS was performed for 30 min at room temperature with or without 2% CHAPS to solubilize the liposomes prior to cross-linking, as indicated. Results were analyzed by immunoblotting. (E) Bax (100 nM) was incubated with or without liposomes for 2 h. Cross-linking and immunoblotting were performed as in (D). (F) Membrane-bound Bcl-XL inhibits the liposome-induced Bax conformational change with 50 μM m1Bid but not with 50 μM Bak BH3 peptide. Immunoprecipitations and immunoblotting were performed as in (B).

We hypothesized that the residual antiapoptotic function of membrane-bound Bcl-XL that does not require stable heterodimerization with tBid or Bax involves inhibiting the transient Bax conformational change that occurs when Bax interacts with the membrane surface and exposes an epitope bound by the 6A7 monoclonal antibody [7]. Binding of 6A7 has been used widely as a marker for one of the stages required for activation of Bax (reviewed in [10]). In vivo, this conformational change has been observed for membrane-bound Bax actively involved in membrane permeabilization [38,39]. However, in experiments using liposomes, it is evident that the exposure of this epitope occurs prior to and is independent of Bax membrane insertion. The change is presumed to result from an interaction of Bax with the surface of the membrane because it rapidly reverses when Bax is separated from the liposomes [7]. This liposome-induced conformational change does not require tBid; however, the epitope remains exposed after tBid-induced Bax membrane insertion (Figure 5A, lanes 1 and 2). Unlike the conformational change that accompanies tBid-induced insertion of Bax into membranes, the liposome-induced conformational change also disappears if liposomes are solubilized in CHAPS prior to immunoprecipitation (Figure 5A, compare lanes 1 and 3).

As shown above, addition of Bcl-XL prevents tBid-induced insertion of Bax into membranes and instead results in Bcl-XL inserting into the membrane (Figures 1 and 4). However, under these conditions the soluble Bax does not undergo the liposome-induced conformation change (Figure 5B), suggesting that the presence of membrane-bound Bcl-XL either inhibits this conformational change or shifts the equilibrium of Bax molecules interconverting between the two forms sufficiently towards the 6A7-negative conformer that the antibody no longer has sufficient access to the epitope. As previously described, soluble Bcl-XL had little effect on the liposome-induced conformational change of Bax (Figure 5C, top panel) [7]. In contrast, the spontaneous membrane binding Bcl-XL Y101K mutant inhibits this conformational change in Bax (Figure 5C, bottom panel).

To investigate further the effects of the membrane surface on Bax and the inhibition of these effects by Bcl-XL, cross-linking experiments using disuccinimidyl suberate (DSS) were performed. Cross-linking of Bax into higher-order structures after Bax binds to membranes has been observed previously [18]. The incubation of Bax with liposomes alone does not cause sufficiently tight membrane binding by Bax to survive gel filtration chromatography (Figure 1B). Nevertheless, incubation with liposomes did result in the cross-linking of Bax into higher-order complexes (Figure 5D, left panel). As expected from previous results [7,9], the interactions between Bax monomers induced by incubation with membranes were not resistant to detergent solubilization prior to cross-linking. The Bax–Bax cross-links were reduced in the absence of liposomes (Figure 5E), suggesting that, similar to binding by the 6A7 antibody, they result from a liposome-induced conformational change in Bax. Addition of tBid to Bax and liposomes resulted in a similar cross-linking pattern, but these Bax oligomers were resistant to solubilization of the membrane with detergent (Figure 5D, middle panel). Membrane-bound Bcl-XL not only prevented the formation of detergent-resistant Bax cross-links but also prevented the cross-linking of Bax that resulted when Bax contacted the membrane surface (Figure 5D, right panel). These results further demonstrate that Bcl-XL in the membrane prevents or reverses the conformational change in soluble Bax that occurs upon exposure to a membrane surface.

To determine whether the effect of membrane-bound Bcl-XL on this transient, liposome-induced conformational change in Bax could be regulated, BH3 peptides were used to induce selectively Bcl-XL binding to membranes (Text S1). On the basis of the effects previously published for mutations in BH3 domains of proapoptotic proteins and peptides, we selected two peptides that both caused membrane binding by Bcl-XL (Figure S5A) but differed in their functional effects (Figure S5B). One peptide, designated m1Bid BH3, containing a single mutation for a conserved and critical leucine residue, did not interfere with the antiapoptotic function of Bcl-XL. The other peptide, Bak BH3, effectively eliminated the antiapoptotic activity of Bcl-XL as assayed by liposome permeabilization assays. The effects of these peptides indicate that membrane-bound Bcl-XL can exist in both functional and nonfunctional states.

To determine whether the functional state of membrane-bound Bcl-XL affected the inhibition of the liposome-induced conformational change in Bax, the m1Bid BH3 and Bak BH3 peptides were used to trigger membrane binding by Bcl-XL in the presence of soluble Bax. In the absence of Bcl-XL, neither peptide induced Bax membrane binding or Bax-dependent membrane permeabilization (unpublished data). When the m1Bid BH3 peptide triggered Bcl-XL binding to membranes, the Bcl-XL still inhibited the liposome-induced Bax conformational change (Figure 5F, top panel, lanes 2 and 3). In contrast, when the Bak BH3 peptide was added, membrane-bound Bcl-XL did not prevent the liposome-induced Bax conformational change (Figure 5F, bottom panel, lanes 2 and 3). Therefore, inhibition of the liposome-induced conformational change in soluble Bax by membrane-bound Bcl-XL correlates with the functional status of Bcl-XL on the membrane. Taken together, these results indicate that this activity is a regulatable function of membrane-bound Bcl-XL and is not merely the result of changes in the biophysical properties of liposomes after Bcl-XL binding (Figure 6D).

## Discussion

To date, it has been a paradox that Bcl-XL and Bax have very similar structures, yet proposed models for their function all suggest that they behave very differently. Taken together, our results suggest that they actually function similarly to the extent that they compete with each other at most steps of the process but with the major difference being that Bcl-XL is defective for membrane permeabilization. Thus, for most functions Bcl-XL behaves in a manner conceptually similar to a dominant-negative Bax. Bcl-XL inhibited membrane binding by Bax by competing with soluble Bax for recruitment to membranes by either tBid or membrane-bound Bax (Figure 6). Thus, similar to Bax, Bcl-XL binds to both activated, membrane-bound Bax and membrane-bound tBid. Furthermore, Bcl-XL binding to Bax inhibits the subsequent oligomerization of membrane-bound Bax. Our results with mutations that disable either of these interactions individually indicate that in membranes both are functionally important.

Pioneering experiments to identify relevant binding partners for Bcl-2 and Bcl-XL using immunoprecipitation in transfected cells suggested a lack of correlation between Bax binding and inhibition of apoptosis, as only certain Bcl-XL point mutants that could no longer bind to Bax lost function [40]. Further analysis of two of these Bcl-XL mutants that did not bind to Bax (the F131V/D133A mutant that remained functional and the inactive G138E/R139L/I140N mutant) suggested that prevention of apoptosis required binding to the BH3-only proteins tBid, Bim, and Bad, a function that was specifically lost in the latter mutant [19]. However, as these experiments were conducted on whole cells where Bax was also present, it is possible that the lack of function of this mutant is caused by the loss of binding to both BH3-only proteins (e.g., tBid) and Bax , a result entirely consistent with our observations. Conversely, early work with the M97A/D98A Bid mutant that does not bind to Bcl-XL [27,30] that we have used in our study (tBid mt1) indicated that Bcl-XL inhibited the apoptosis caused by this Bid mutant, in cells and in purified mitochondria, implying that the interaction with activated Bax rather than Bid was critical to the antiapoptotic function of Bcl-XL in this context. Our model reconciles these disparate results by postulating that only after loss of both interactions does the antiapoptotic function of Bcl-XL become severely diminished. While Bcl-XL may inhibit OMM permeabilization initiated by the BH3-only proteins Bid, Bim, and Puma by sequestering these proteins, a variety of evidence now exists for alternate Bax activation pathways (reviewed in [10]) that do not rely on these BH3-only proteins. For many of these known and potentially unknown pathways, Bcl-XL may not directly sequester the initial activators of Bax, and hence inhibition of membrane permeabilization may rely on the sequestration of activated Bax to inhibit Bax autoactivation, oligomerization, and membrane permeabilization.

Our results clearly show that the major functional interactions of Bcl-XL occur after the protein has migrated to the membrane, a process initiated in our in vitro system by binding to either membrane-localized tBid or membrane-localized (activated) Bax. Membrane insertion of both tBid [41] and Bax [42] exposes the proapoptotic BH3 region of each protein, an event that is critical for interaction with Bcl-XL and likely initiates a conformational change in the antiapoptotic protein that is required for membrane insertion. Bcl-XL is found in both the cytoplasm as well as attached to (but not inserted into) the mitochondrial membrane in healthy cells. Migration of the cytoplasmic fraction to membranes with insertion occurs during apoptosis [23,43,44] for Bcl-XL as well as other antiapoptotic proteins such as Mcl-1 [44] and Bcl-w [25,45]. For Bcl-w, interaction with a tethered Bim BH3 peptide displaces the C-terminal insertion sequence from the BH3 binding pocket within the protein triggering insertion of the protein into the membrane [25,46]. Although a structure has not been reported for Bcl-XL containing the C-terminal insertion sequence, the structure of the truncated protein is sufficiently similar to that of Bcl-w [46] and proapoptotic Bax [36] to suggest displacement of the insertion sequence of Bcl-XL as a mechanism that drives Bcl-XL into the membrane. In vitro binding assays using protein lacking the C-terminal insertion sequence have shown that Bcl-XL binds a variety of BH3 peptides [2,34]. For several of the peptides, structural studies have confirmed that the peptides bind in the hydrophobic pocket that is also believed to bind the Bcl-XL insertion sequence [47–49]. Furthermore, it has been reported previously that overexpression of Bad caused the membrane insertion of Bcl-XL in HeLa cells [33]. We therefore determined whether peptides from the BH3 regions of Bid, Bim, Bad, Bax, and Bak caused insertion of Bcl-XL into liposomes, assayed by flotation on a sucrose gradient (Figure S6). All five peptides caused Bcl-XL to insert into membranes, while a mutant Bid peptide that fails to bind Bcl-XL [2] did not.

In cells, it is likely that other BH3-only proteins will substitute in many cases for tBid, and unlike Bid some of these proteins may not be required themselves to bind to membranes to expose the BH3 sequence. Nevertheless, whether initiated in the cytoplasm or at the surface of the membrane, interaction of Bcl-XL with a proapoptotic BH3 sequence is likely to be at least one of the factors that contributes to the insertion of Bcl-XL into membranes.

Previous reports using transfected cells have indicated that the removal of the C-terminal insertion sequence of Bcl-XL severely impairs membrane insertion but has varying effects on Bcl-XL function, from moderate [29] to severe [50] reduction in function. Consistent with this variability, we have shown previously [37] that the function of ΔTM Bcl-XL compared to that of wild type is heavily dependent on the stimulus used to initiate apoptosis (and therefore possibly the BH3 protein(s) involved). In our in vitro system, ΔTM Bcl-XL showed a severe loss of function (Figure S3) as the relevant interactions here occur on membranes. However, in certain circumstances in cells ΔTM Bcl-XL may retain at least part of its antiapoptotic function in the cytoplasm depending on the location of relevant binding partners at the onset of apoptosis.

While we have focused our experiments on examining tBid and its interactions with Bax and Bcl-XL, our studies do not imply that tBid is an essential component of the membrane permeabilization process. Instead, we predict that various aspects of the functions of Bcl-XL and Bax revealed here can be triggered differentially by the different BH3 proteins that have been identified. Moreover, different BH3-only proteins would be expected to exhibit differences in the relative affinities for and activities on Bax and Bcl-XL, as we have described in our “embedded together” model [10]. However, the previously reported binding affinities between BH3 peptides and other Bcl-2 family members are unlikely to accurately reflect binding affinities at the physiologically relevant locus of membranes. Clearly, it will be important to determine more appropriate quantitative estimates for the interactions of Bcl-2 family proteins in membranes.

Inhibition of the transient conformation change in Bax that occurs at the membrane surface (Figure 6D) is the only step in which inhibition of Bax activation is not due to Bcl-XL functioning similar to a defective version of Bax. It is likely that the conformational change in Bax at the membrane surface enhances its activation by membrane-bound tBid. Consistent with this interpretation, tethering of the soluble Bid BH3 peptide to liposomes increased its potency by several orders of magnitude [51]. It is not known how membrane-bound Bcl-XL prevents a transient change in the conformation of Bax. However, it is not unreasonable to speculate that it might do so by interacting transiently with Bax to shift the equilibrium in Bax conformers sufficiently toward the 6A7-negative conformation that the antibody does not have sufficient access to the epitope to bind it.

Our in vitro system recapitulates the core features of organelle permeabilization by activated Bax and its inhibition by Bcl-XL and has allowed us to identify and examine many of the individual steps. The liposomes that we used to model physiologic membranes have a high intrinsic curvature and lipid composition that facilitate membrane binding of the recombinant proteins and induction of the 6A7 conformational change in Bax. In cells, these feature are likely represented by complex and dynamic physiologic processes such as mitochondrial membrane fission and fusion shown to be important in apoptosis [52–54] and the interaction of mitochondria with other membrane systems [39,52,55]. The major observations reported here were confirmed for cytochrome c release from mitochondria (Figure 3B–D), suggesting that they will be relevant in live cells. Moreover, the simplicity and power of our in vitro system using recombinant proteins and membranes has allowed us to identify and measure functionally important and potentially “druggable” interactions important for the regulation of apoptosis (Figure 6).

## Materials and Methods

### Materials.

The *bak* –/– mice were purchased from Jackson Laboratories. ANTS and DPX were purchased from Molecular Probes. BH3 peptides blocked at both ends (Ac-peptide-amide) were obtained from Dalton Chemicals. The monoclonal Bax antibodies 2D2 and 6A7 were generous gifts from Richard Youle [26]. The monoclonal tBid antibody 5C8 was obtained from Exalpha Biologicals. The rabbit polyclonal antibody to Bcl-XL and the sheep polyclonal antibody to cytochrome c were produced in our laboratory. Immunoblotting of Bax was carried out using 2D2 at a dilution of 1:10,000. Immunoblotting of Bcl-XL, tBid, and cytochrome c was performed at dilutions of 1:10,000, 1:2,000, and 1:5,000, respectively, with the appropriate antibody. Secondary antibodies conjugated to horseradish peroxidase were purchased from Jackson Immuno Research Laboratories and were used at dilutions of 1:10,000. Immunoblots were analyzed using ImageQuant (version 5.2, Molecular Dynamics). Statistical analysis was performed using a one-way ANOVA model. All lipids were obtained from Avanti Polar Lipids. DSS was purchased from Pierce.

### Protein purification.

Recombinant full-length human Bcl-XL (or Bcl-XL Y101K) with no additional amino acids was expressed in Escherichia coli as a C-terminal intein/chitin-binding domain fusion and purified by affinity chromatography on a chitin column followed by further purification on a phenyl-Sepharose column, similar to a method described previously [7] but with a final dialysis step to remove detergents. For ΔTM Bcl-XL (and the ΔTM Y101K mutant), the phenyl-Sepharose chromatography step was omitted. Recombinant full-length human Bax and murine tBid (or tBid-mt1) with no additional amino acids were purified as described previously [7,9].

### Membrane preparation and measurement of permeabilization.

Liposomes were composed of the following molar percentages of lipids: phosphatidylcholine, 48%; phosphatidylethanolamine, 28%; phosphatidylinositol, 10%; dioleoyl phosphatidylserine, 10%; and tetraoleoyl cardiolipin, 4%. Liposome preparation, including ANTS/DPX charged liposomes, was essentially as in [7], with the exception that liposomes were prepared in assay buffer (10 mM HEPES (pH 7), 200 mM KCl, 5 mM MgCl_2_, and 0.2 mM EDTA). Samples (50 μM total lipids) were prepared in assay buffer with all sample components (buffers, liposomes, etc.) added prior to the addition of recombinant proteins. Fluorescence (λ_ex_ = 355 nm and λ_em_ = 520 nM) was measured for 30 min at 37 °C in the presence of Bcl-XL but in the absence of Bax and tBid to obtain background values (*F*_0_). Bax and tBid (in that order) were added at *t* = 0, and fluorescence was measured for 2 h at 37 °C. Triton X-100 was added to a final concentration of 0.2% (w/v), and fluorescence was measured for 10 min at 37 °C (*F*_100_). The percentage release of ANTS/DPX was calculated as percentage release = ((*F* – *F*_0_)/(*F*_100_ – *F*_0_)) × 100.

Membrane fractions containing mitochondria from C57bl6 mouse liver were purified as described previously [56]. Samples were diluted to 1 mg/ml total protein (Bradford assay), incubated with purified proteins, added in the order Bcl-XL, Bax, and tBid, for 1 h at 30 °C, then centrifuged at 13,000 *g* for 10 min. The supernatant and pellet were separated and analyzed.

### Membrane binding assays.

Samples containing liposomes (300 μM total lipids) were prepared in assay buffer and incubated at 37 °C for 2 h. All sample components (buffers, liposomes, etc.) were added prior to the addition of recombinant proteins, which were added in the order Bcl-XL, Bax, and tBid. Membrane-bound protein was separated from soluble (“free”) protein using gel filtration chromatography on Sepharose CL-2B resin. Membrane binding was measured by comparing the intensities of membrane-bound proteins (fractions 3 and 4) with total proteins (fractions 3 and 4 plus fractions 8–11). Separation of membrane-bound protein from soluble (“free”) protein by liposome floatation on a sucrose density gradient was performed as previously described [57]. To assess the membrane binding and membrane insertion of Bax and Bcl-XL into mitochondria, purified proteins, added in the order Bcl-XL, Bax, and tBid, were incubated for 1 h at 30 °C with mitochondria (5 mg/ml) from *bak* –/– mice. Mitochondria were centrifuged at 7,000 *g* for 10 min. Pellets were resuspended (50 μl) and treated with 800 μl of carbonate buffer (200 mM sodium carbonate (pH 11.5), 10 mM DTT, and 2% glycerol) for 30 min on ice. Samples (750 μl) were then overlayed onto a 0.5 M sucrose cushion in carbonate buffer (250 μl) and centrifuged at ∼200,000*g* for 30 min. The supernatant and sucrose cushion were separated, neutralized with glacial acetic acid (5 μl per 250 μl sample), and stored at −80 °C, or the proteins were precipitated with trichloroacetic acid (18.75%). All immunoblots are representative of at least three independent experiments.

### Immunoprecipitation.

Samples containing liposomes (300 μM total lipids) were prepared and incubated as described in the “Membrane binding assays” section. Immunoprecipitation of Bcl-XL was performed using the polyclonal Bcl-XL antibody in assay buffer containing either 2% CHAPS or 0.2% NP-40. Immunoprecipitates were collected as previously described [7] and washed three times in assay buffer containing the appropriate detergent. Immunoprecipitation using the conformation-specific 6A7 Bax antibody was performed on whole membranes and washed three times with assay buffer containing 2% CHAPS. All immunoblots are representative of at least three independent experiments.

### DSS cross-linking.

Samples containing liposomes (300 μM total lipids) were prepared and incubated as described in the “Membrane binding assays” section. DSS cross-linking was performed at a concentration of 2 mM (or DMSO control) for 30 min at room temperature. The cross-linker was quenched by the addition of Tris-Cl (pH 8) to a final concentration of 20 mM. To examine the effects of CHAPS solubilization prior to cross-linking, samples were mixed with an equal volume of assay buffer or assay buffer containing 4% CHAPS, and the extent of cross-linking was analyzed. All immunoblots are representative of at least three independent experiments.

## Supporting Information

Figure S1 Bax and tBid Cooperate To Induce Liposome Permeabilization Allowing Egress of 10 kDa Dextran, Which Is Inhibited by Bcl-XL (A) Liposomes (300 μM total lipids) containing a rhodamine-PE tracer (0.1% of total lipids) encapsulating Bodipy-labeled 10 kDa dextrans (30 μM) were incubated with 100 nM Bax, 20 nM tBid, and the indicated amounts of Bcl-XL. Liposomes were separated from released dextrans by Sepharose CL-2B gel filtration chromatography. Bodipy (λ_ex_ = 460 nm and λ_em_ = 518 nm, left panel) and rhodamine (λ_ex_ = 590 nm and λ_em_ = 610 nm, right panel). Excluded fractions (3–6) contain liposomes and encapsulated dextrans. Included fractions (8–13) contain dextrans released from liposomes. Fluorescence was expressed as a percentage of the total.
(B) Bcl-XL inhibits liposome permeabilization competitively. Liposomes (50 μM total lipids) encapsulating ANTS and DPX were incubated with 100 nM Bax, 20 nM tBid, and the indicated concentration of Bcl-XL. Membrane permeabilization was assayed by an increase of ANTS fluorescence and presented as mean ± standard deviation for at least three independent experiments. The IC_50_ value (25.5 nM) is indicated by the dashed line and was calculated using Graphit software (version 1.10, Gecces Software Development).
(392 KB TIF)

Figure S2 Residual Binding of Bcl-XL to tBid-mt1 and Bcl-XL Y101K to Bax (A) Bax (100 nM) and 20 nM tBid (left) or tBid-mt1 (right) were incubated with 20 or 100 nM Bcl-XL and liposomes. Samples were immunoprecipitated (IP) in 2% CHAPS, using an antibody with the indicated specificity, and immunoblotted (IB) for the indicated protein. Normal (“light”) exposures are shown at the top while excessive (“dark”) exposures are shown at the bottom. Input panels represent 3% of the entire reaction while immunoprecipitation panels represent 20% of the entire reaction.
(B) Bax (100 nM) and tBid-mt1 (20 nM) were incubated with 20 or 100 nM Bcl-XL (left) or Bcl-XL Y101K (right) and liposomes. Immunoprecipitations and immunoblotting were performed as in (A), with input panels representing 1% of the entire reaction and immunoprecipitation panels representing 20% of the entire reaction.
(615 KB TIF)

Figure S3 Bcl-XL Binds to Bax in Membranes, Inhibiting Bax Oligomerization and Membrane Permeabilization (A) Bax, tBid-mt1, and Bcl-XL (as indicated) were incubated with liposomes (300 μM total lipids). The membrane fraction was isolated by Sepharose CL-2B gel filtration chromatography and immunoblotted for Bax (top panel) or Bcl-XL (second panel). Cross-linking with DSS (2 mM) was performed for 30 min at room temperature on whole membranes and analyzed by immunoblotting for Bax (third panel). When Bcl-XL is present, there is a large increase in the amount of monomer and a decrease in the amount of cross-linked species most noticeable for trimers and above. Bax and Bcl-XL coprecipitated (fourth and fifth panels) when membrane fractions were immunoprecipitated with either antibody (indicated to the right of the panels) and immunoblotted with the opposite antibody (indicated to the left of the panels). The asterisk indicates the antibody light chain.
(B) Liposomes (50 μM total lipids) encapsulating ANTS and DPX were incubated with Bax, tBid-mt1, and Bcl-XL (as indicated). Membrane permeabilization was assayed by an increase of ANTS fluorescence.
(425 KB TIF)

Figure S4 Loss of Membrane Binding Inhibits the Residual Function of a Heterodimerization-Incompetent Bcl-XL (A) Bcl-XL or Bcl-XL Y101K lacking 23 C-terminal residues (ΔTM Bcl-XL or ΔTM Bcl-XL Y101K) (100 nM) were incubated with liposomes (300 μM total lipids), 100 nM Bax, and 20 nM tBid or tBid-mt1. Membrane-bound protein was separated from free protein by Sepharose CL-2B gel filtration chromatography. Individual fractions were analyzed by immunoblotting for Bcl-XL.
(B) Liposomes (50 μM total lipids) encapsulating ANTS and DPX were incubated with 100 nM Bax, 20 nM tBid or tBid-mt1, and increasing concentrations of ΔTM Bcl-XL, ΔTM Bcl-XL Y101K, or Bcl-XL Y101K. Membrane permeabilization was assayed by an increase of ANTS fluorescence and is presented as mean ± standard deviation for at least three independent experiments.
(199 KB TIF)

Figure S5 Membrane-Bound Bcl-XL Can Be in a Functional or Nonfunctional State (A) Membrane binding of Bcl-XL can be triggered by BH3 peptides. Left, sequences of peptides corresponding to the BH3 regions of Bid, a Bcl-XL-binding mutant m1Bid, an inactive mutant m2BidBH3, and Bak. Right, 100 nM Bcl-XL incubated with liposomes (300 μM total lipids) and the indicated BH3 peptide (50 μM each). Membrane-bound protein was separated from soluble protein by Sepharose CL-2B gel filtration chromatography, and individual fractions were analyzed by immunoblotting for Bcl-XL.
(B) Membrane-bound Bcl-XL is functional (left panel) or nonfunctional (right panel) when incubated with m1Bid BH3 or Bak BH3 peptide, respectively. Liposomes (50 μM total lipids) encapsulating ANTS and DPX were incubated with the indicated concentration of Bcl-XL and m1Bid BH3 peptide (left panel) or Bak BH3 peptide (right panel) for 2 h prior to the addition of 100 nM Bax and 20 nM tBid. Membrane permeabilization was assayed by an increase of ANTS fluorescence, and results are representative of at least three independent experiments.
(1.0 MB TIF)

Figure S6 BH3 Peptides Induce Binding of Bcl-XL to Membranes (A) Sequences of peptides corresponding to the BH3 regions of Bid, an inactive mutant m2Bid, Bim, Bad, Bax, and Bak.
(B) Bcl-XL (100 nM) was incubated with liposomes (300 μM total lipids) and the indicated BH3 peptide (50 μM each). Membrane-bound protein was separated from soluble protein by liposome floatation on a discontinuous sucrose gradient. Individual fractions were analyzed by immunoblotting for Bcl-XL.
(569 KB TIF)

Text S1 Membrane-Bound Bcl-XL Can Exist in Both Functional and Nonfunctional States (36 KB DOC)

## Abbreviations

ANTS: 8-aminonaphthalene 1,3,6-trisulfonic acid
DPX: *p*-xylene-bis-pyridinium
DSS: disuccinimidyl suberate
MLM: mouse liver mitochondria
OMM: outer mitochondrial membrane
tBid: caspase-8 cleavage product of Bid
