# Supplementary material for: Bcl-XL Inhibits Membrane Permeabilization by Competing with Bax
Source: PLoS Biol. 2008 Jun 10;6(6):e147. doi: 10.1371/journal.pbio.0060147 (PMC2422857; doi:10.1371/journal.pbio.0060147)
Supplement: Supplementary file 1 [file pbio.0060147.sd001.doc]

**Membrane bound Bcl-XL can exist in both functional and non-functional states**

To determine whether we could induce Bcl-XL membrane binding in both functional and non-functional states, we evaluated several BH3 peptides which had activity specific to Bcl-XL in our model system. Unlike Bid BH3, m1Bid BH3 does not induce membrane permeabilization in the presence of Bax (data not shown). Furthermore, mutation of this residue has been shown to severely impair the affinity of BH3 peptides for anti-apoptotic proteins [1-3]. However, m1Bid BH3 still induced Bcl-XL membrane binding similar to Bid BH3 (Supplementary Figure 5A). Further mutation of a conserved apartate residue (m2Bid BH3) completely abolishes the affinity of this peptide for Bcl-XL [4] and hence does not induce Bcl-XL membrane binding. The second peptide, Bak BH3, also does not induce membrane permeabilization in the presence of Bax (data not shown). However, this peptide has a high affinity for Bcl-XL [1] and also induces Bcl-XL membrane binding. When these two peptides were used to induce Bcl-XL membrane binding prior to the addition of tBid and Bax, Bcl-XL treated with m1Bid BH3 remained largely functional, while Bcl-XL treated with Bak BH3 was completely non-functional (Supplementary Figure 5B).

**References**

1. Sattler M, Liang H, Nettesheim D, Meadows RP, Harlan JE, et al. (1997) Structure of Bcl-xL-Bak peptide complex: recognition between regulators of apoptosis. Science 275: 983-986.

2. Uren RT, Dewson G, Chen L, Coyne SC, Huang DC, et al. (2007) Mitochondrial permeabilization relies on BH3 ligands engaging multiple prosurvival Bcl-2 relatives, not Bak. J Cell Biol 177: 277-287.

3. Ruffolo SC, Shore GC (2003) BCL-2 selectively interacts with the BID-induced open conformer of BAK, inhibiting BAK auto-oligomerization. J Biol Chem 278: 25039-25045.

4. Certo M, Del Gaizo Moore V, Nishino M, Wei G, Korsmeyer S, et al. (2006) Mitochondria primed by death signals determine cellular addiction to antiapoptotic BCL-2 family members. Cancer Cell 9: 351-365.
